# The Price of Glass Eyes in England

**Published:** 1894-06

**Authors:** 


### The Price of Glass Eyes in England.

The best glass eyes are made in Paris, where patients are generally sent who are anxious for a perfect match; but excellent ones, at a much lower price, are made in Birmingham; and if care is exercised in the choice, a perfect match can generally be obtained. Lately German eyes have been supplied to many hospitals, costing wholesale only about 2s. 6d. each. A stock of three hundred of these is sufficient for ordinary work, and they can be supplied to poor patients for four or five shillings each—an immense blessing, as many a mill girl and factory hand can testify.—*London Tit-Bits.*

